# Determinants of diabetes knowledge in a cohort of Nigerian diabetics

**DOI:** 10.1186/2251-6581-13-39

**Published:** 2014-03-04

**Authors:** Unyime Sunday Jasper, Babatunde Gbolahan Ogundunmade, Macmillian Chinonso Opara, Olayinka Akinrolie, Edna Bawa Pyiki, Aishatu Umar

**Affiliations:** 1 Department of Physiotherapy, Jos University Teaching Hospital, Jos, Nigeria; 2 Department of physiotherapy, National Hospital, Abuja, Nigeria

**Keywords:** Determinants, Knowledge, Sociodemographic, Diabetes, Nigeria

## Abstract

**Background:**

One of the consequences of the generational paradigm shift of lifestyle from the traditional African model to a more "western" standard is a replacement of communicable diseases by non-communicable or life style related diseases like diabetes. To address this trend, diabetes education along with continuous assessment of diabetes related knowledge has been advocated. Since most of the Nigerian studies assessing knowledge of diabetes were hospital-based, we decided to evaluate the diabetes related knowledge and its sociodemographic determinants in a general population of diabetics.

**Methods:**

Diabetics (n = 184) attending the 2012 world diabetes day celebration in a Nigerian community were surveyed using a two part questionnaire. Section A elicited information on their demographics characteristics and participation in update courses, and exercise, while section B assessed knowledge of diabetes using the 14 item Michigan Diabetes Research and Training Centre's Brief Diabetes Knowledge Test.

**Results:**

We found that Nigerian diabetics had poor knowledge of diabetes, with pervasive fallacies. Majority did not have knowledge of "diabetes diet", "fatty food", "free food", effect of unsweetened fruit juice on blood glucose, treatment of hypoglycaemia, and the average duration glycosylated haemoglobin (haemoglobin A1) test measures blood glucose. Attaining tertiary education, falling under the 51-60 years age group, frequent attendance at seminars/updates and satisfaction with education received, being employed by or formerly working for the government, and claiming an intermediate, or wealthy income status was associated with better knowledge of diabetes.

**Conclusion:**

Nigerian diabetics' knowledge of diabetes was poor and related to age, level of education, satisfaction with education received, employment status and household wealth.

## Background

Diabetes is projected to become one of the world's main disabler and killer within the next twenty-five years [1] and the developing countries in Africa are not left out. This can be attributed to advancement in education and technology, coupled with increase in urbanization and exchange of ideas between the developed and developing countries. The resultant effect is a continuous generational paradigm shift of lifestyle from the customary African model to a more “western” standard. One of the consequences of this transition is a change in disease patterns with communicable diseases being replaced by non-communicable or life style related diseases like diabetes, obesity, cardiovascular disease and cancer [2]. This is because the healthier conventional lifestyles which was characterised by regular and vigorous physical activity accompanied by sustenance on high fibre whole grain-based diet, rich in vegetables, and fruits^2^ has been replaced by over-reliance on motorised transport and consumption of unhealthy diets rich in carbohydrates, fats, sugars, and salts [3]. The resultant effects of this “adopted” regime are an upsurge in the levels of obesity and overweight in the population, itself a risk factor for diabetes.

The sole greatest panacea and deterrent against diabetes is adequate knowledge of the condition. It has been reported that information can help people assess their risk of diabetes, motivate them to seek proper treatment and care, and inspire them to take charge of their disease [4,5]. Knowledge of diabetes forms the basis for informed decisions about diet, exercise, weight control, blood glucose monitoring, use of medications, foot and eye care, and control of macro vascular risk factors [6]. Knowledge and awareness about DM, its risk factors, complications, and management are important aspects for better control and better quality of life [7,8]. As diabetes mellitus is a chronic disease, adherence to appropriate self-care practices leads to improved glycaemic control. Furthermore, Ranjini et al. [9] showed that more knowledgeable diabetic patients had better attitude towards the care of their own disease. If proper education is incorporated into a structured diabetic care programme in health care settings, more value will be added to patients' knowledge and self-care behaviour.

In Nigeria, a substantial number of studies have assessed knowledge of the causes, risk factors, complications, and management of diabetes among diabetic patients [10-12], diabetic and non diabetics [13], and to evaluate the effect of an educational intervention on diabetes knowledge 21 [14]. While some studies reported poor knowledge [11,12], another reported good to fair knowledge [10,13] and Puepet et al. [14] found that educational programme impacts positively on the knowledge of diabetes. However, most of these studies were hospital based and may therefore not have assessed other diabetes patients who for one reason or another do not visit the hospital regularly or go to a diabetes centre for check up or follow up. Therefore the aim of this study was to assess diabetes knowledge and its sociodemographic determinants among a general population of diabetes patients.

## Material and methods

### Sample

This study was a descriptive cross-sectional study which utilized a sampling of convenience to recruit all eligible diabetic patients who attended the 2012 world diabetes day celebration at a diabetes screening centre in Jos, Plateau State, Nigeria.

### Instrument

The instrument utilized in this study was a two-part questionnaire. Section A dealt with patient demographics (age, gender, religion, level of education, occupation/employment status, level of household wealth and family history of diabetes etc) and the disease (time since diagnosis, type of treatment, regularity on medication and type of diabetes). It also contains questions on their involvement in exercise, whether exercise is beneficial for diabetes, reading/attending update courses and satisfaction with information gathered. Section B assessed basic knowledge of diabetes mellitus using the Michigan Diabetes Research and Training Centre’s Brief Diabetes Knowledge Test, which was created for adults with either type 1 or type 2 diabetes [15]. Fourteen multiple-choice questions assess basic patient knowledge of diabetes, while nine assess patient’s knowledge of insulin use. A reliability coefficient of 0.70 was reported for the general knowledge subscale [15].

### Methodology

Approval to carry out this study was sought and obtained from the management of the diabetes screening centre in Jos, Plateau State, Nigeria. Prior to the commencement of the interview, the purpose of the study was thoroughly explained to the participants. All the participants who had a good grasp of English language or who could understand the contents of the questionnaire when it was explained to them, and were willing to participate in the study were assessed. Furthermore the researchers were on hand to attend to any questions arising from the respondents, while they assisted those who could neither read nor write to complete their questionnaires.

It was ensured that the feedback came from them so as to ensure that they understood the questions very well. For those with visual impairments, the questions were read out to them and they provided answers. However, participants who have not previously been diagnosed of diabetes were excluded from the study as were those who could not understand the contents of the questionnaire after it has been explained to them (illiterate or visually impaired). Participants who were under 18 years, mentally or speech impaired were also excluded.

Participants who are on other medications apart from insulin were required to answer questions 1-14 (general knowledge of diabetes test) on section B of the questionnaire [15]. A score of ≥ seven was considered satisfactory in this study. Each correct answer was awarded one point and the total score was rated as good (>7), or poor knowledge (<7), with the maximum score obtainable being 14. Higher scores indicate higher knowledge of diabetes.

### Data analysis

Using SPSS version 17, descriptive statistics of percentages was computed for the sociodemographic variables, previous education, satisfaction with education, involvement in regular exercise, knowledge of benefit of exercise and correct response to each question in section B. Analysis of variance (ANOVA) and independent t-test was used to determine the influence of sociodemographic variables on knowledge of diabetes. Proportional differences were explored using chi statistics. Differences were considered significant at an alpha level of 0.05.

## Results

Out of the two hundred and fifteen (215) questionnaires distributed, 202 were returned and 18 were considered invalid, translating to a response rate of 85.5% (n = 184). A simple majority of the participants in this study were between the age group of 51-60 years (34.2%, n = 63), while those between 71-80 years respectively represented the least age group (4.3%, n = 8). Further sociodemographic information is shown in Table 1.

**Table 1:** Sociodemographic variables of participants

**Demographic variables**	**n**	**%**	***X***^***2***^	**p-value**
**Age**				
21-30 years	13	7 · 1		
31-40 years	35	19 · 1		
41-50 years	32	17 · 3	108 · 9	0.000
51-60 years	63	34 · 2		
61-70 years	33	17 · 9		
71-80 years	8	4 · 3		
**Gender**				
Male	68	37 · 0		
Female	116	63 · 0	12 · 5	0 · 000
**Religion**				
Christianity	147	79 · 9		
Islam	37	20 · 1	65 · 8	0 · 000
**Marital status**				
Single	9	4 · 9		
Married	148	80 · 4		
Divorced/Separated	3	1 · 6	306 · 7	0 · 000
Widow/Widower	24	13 · 0		
**Level of education**				
Primary	35	19 · 0		
Secondary	44	23 · 9		
Tertiary	76	41 · 3	28 · 5	0 · 000
None	29	15 · 8		
**Employment status**				
Unemployed	42	22 · 5		
Self employed	44	23 · 9		
Government employed	57	31 · 0	39 · 1	0 · 000
Retired	35	19 · 0		
Student	6	3 · 3		
**Level of household wealth**				
Poor	39	21 · 2		
Intermediate	129	69 · 0	109 · 1	0 · 000
Wealthy	18	9 · 8		
**Do you have a family history of diabetes?**				
Yes	95	51 · 6		
No	60	32 · 6	35 · 6	0 · 000
Not sure	29	15 · 8		
**When were you diagnosed of diabetes**				
Less than 5 years	80	43 · 5		
5-10 years	71	38 · 6		
10-15 years	18	9 · 8	202 · 5	0 · 000
15-20 years	15-213	15-22 1 · 6	15-23	15-24
25-30 years	4	2 · 2		
>30 years	8	4 · 3		
**Type of diabetes**				
Type 1	60	32 · 6	22 · 3	0 · 000
Type 2	124	67 · 4		
**Are you currently on insulin?**				
Yes	56	30 · 4	28 · 2	0 · 000
No	128	69 · 6		
**Where were you diagnosed of diabetes?**				
Private hospital/laboratory	60	32 · 6		
Public hospital	96	52 · 2	37 · 7	0 · 000
Diabetes centre	28	15 · 2		
**Regular on medication**				
Yes	146	79 · 3	286 · 4	0 · 000
No	38	20 · 7		
**Exercise regularly**				
Yes	87	47 · 3	0 · 54	1 · 857
No	97	52 · 7		
**Exercise is beneficial for diabetes**				
Yes	140	76 · 1		
No	6	3 · 3	159 · 7	0 · 000
Not sure	38	20 · 6		
**Read articles/Attend update courses**				
Not at all	56	30 · 4		
Rarely	60	32 · 6		
Often	43	23 · 4	16 · 2	0 · 976
Regularly	25	13 · 6		
**Satisfied with instruction**				
Very satisfied	20	10 · 8		
Satisfied	75	40 · 8	33 · 2	0 · 000
Not satisfied	89	48 · 4		

### Knowledge of diabetes

Even though a simple majority (56.5%, n = 104) had a good knowledge of diabetes, the overall mean knowledge score (6.2 ± 2.2) was poor. An overwhelming majority did not know that a “free food” is any food that has less than 20 calories per serving (88.0%, n = 162); glycosylated haemoglobin A1 (HbA1c) is a test used to measure the average blood glucose level for the past 6-10 weeks (85.3%, n = 157). The most commonly missed questions are shown in Table 2.

**Table 2:** Most commonly missed questions on the diabetes knowledge test

**Item**	**% Incorrect**	**Question (correct answer is in bold)**
1.	50.5	The diabetes diet is?
		a. The way most American people eat
		**b. A healthy diet for most people**
		c. Too high in carbohydrate for most people
		d. Too high in protein for most people
2.	67.9	Which of the following is highest in carbohydrate?
		a. Baked chicken
		b. Swiss cheese
		**c. Baked potato**
		d. Peanut butter
3.	45.7	Which of the following is highest in fat?
		a. **Low fat milk**
		b. Orange juice
		c. Corn
		d. Honey
4	88.8	Which of the following is a “free food”?
		a. Any unsweetened food
		b. Any dietetic food
		c. Any food that says “sugar free” on the label
		**d. Any food that has less than 20 calories per serving**
5	85.3	Glycosylated haemoglobin (haemoglobin A1) is a test that is a measure of your average blood glucose level for the past:
		a. day
		b. week
		**c. 6–10 weeks**
		d. 6 months
7	78.8	What effect does unsweetened fruit juice have on blood glucose?
		a. Lowers it
		**b. Raises it**
		c. Has no effect
8	80.4	Which should not be used to treat low blood glucose?
		a. 3 hard candies
		b. 1/2 cup orange juice
		**c. 1 cup diet soft drink**
		d. 1 cup skim milk
13	62.0	Numbness and tingling may be symptoms of
		a. Kidney disease
		**b. Nerve disease**
		c. Eye disease
		d. Liver disease

### Sociodemographic determinants of diabetes knowledge

Findings in this study reveal that knowledge significantly increases exponentially as level of education attained (*p = 0.000*, F = 19.2), with those who had not attended school having the lowest scores (4.3 ± 2.2), while those with tertiary education scored highest (7.2 ± 1.2). How often a participant read articles/attended update seminars had a significant effect on knowledge (*p = 0.000*, F = 27.1), with participants who updated their knowledge regularly scoring higher (7.8 ± 1.2) than those who did so often (7.3 ± 1.7), rarely (6.2 ± 1.7) and not at all (4.6 ± 2.1). Furthermore those who were either satisfied (7.6 ± 0.9) or very satisfied (7.5 ± 1.8) with education received had better knowledge than those who were not satisfied with education received (4.6 ± 1.9) (*p = 0.000*, F = 87.0). Table 3 depicts the sociodemographic determinants of diabetes knowledge.

**Table 3:** Sociodemographic determinants of diabetes knowledge

**Variables**	**Mean (SD)**	**F-value**	**p-value**
**Level of education attained**			
Primary	5.5 (1.7)^b^		
Secondary	6.0 (2.5)^b^	19.2	0.000
Tertiary	7.2 (1.2)^a^		
None	4.3 (2.2)^b^		
**Read articles/Attend update seminar**			
Regularly	7.8 (1.2)^a^		
Often	7.3 (1.7)^a^	27.1	0.000
Rarely	6.2 (1.7)^b^		
Not at all	4.6 (2.1)^b^		
**Satisfaction with education received**			
Very satisfied	7.5 (1.9)^a^		
Satisfied 7.6 (0.9)^a^	87.0	0.000	
Not satisfied	4.6 (1.9)^b^		
**Employment status**			
Government employed	6.8 (1.5)^a^		
Self employed	5.4 (2.4)^b^	4.7	0.031
Unemployed	5.5 (2.3)^b^		
Retired	6.9 (2.0)^a^		
Student	6.5 (2.6)^a^		
**Age**			
21-30 years	5.2 (2.0)^b^		
31-40 years	5.8 (1.9)^b^		
41-50 years	6.0 (2.2)^b^	2.3	0.046
51-60 years	6.8 (2.1)^a^		
61-70 years	5.6 (2.3)^b^		
71-80 years	6.8 (1.7)^a^		
**Level of household wealth**			
Poor	5.3 (2.1)^b^		
Intermediate	6.3 (2.1)^a^	5.1	0.029
Wealthy	7.0 (2.1)^a^		

For a particular variable, scheffe post hoc test revealed that means with different superscript are significant at p < 0.05 (superscripts a and b). Means that share the same superscript are not significantly different from each other (p > 0.05).

Knowledge of diabetes was not associated with gender, religious afffiliation, having a family history of diabetes, duration since diagnosis, type of diabetes and where they were diagnosed of diabetes (*p > 0.05*).

## Discussion

With the increase in diabetes toward an epidemic dimension, diabetic patients need to be furnished with sufficient and all encompassing knowledge of this condition so as to ensure optimal self-management. This is especially pertinent in the African setting where people tend to hold tenaciously unto time-honoured beliefs about diseases such as diabetes and invariably search for treatment or cure within this traditional setup.

Even though a slight majority (56.5%) of the diabetes patients in this study had satisfactory knowledge scores, the overall mean knowledge score was poor (6.2 ± 2.2) thus explaining the misconceptions depicted in their answers. Majority (88.0%) did not know what a “free food” is, a figure higher than the 58% reported by Murata et al. [6] among diabetic veterans in US, but lower than 98% found in another Nigerian study [12]. This is probably due to difference in geography, and also because of the widespread long-established misconception in Nigeria that any “sugar free” or “unsweetened” food is the ideal meal for diabetic patients. Moreover, this traditional belief may have been responsible for their ignorance that “unsweetened fruit juice” raises blood glucose (78.2%), a comparable result to the 73% reported by Odili, Isiboge and Eregie [12], but lower than 35% in another study [6].

In the present study, only 14.7% knew the relevance of the HbA1c test. A finding lower than the 44% reported by Murata et al. [6], but in the range of 11% reported in Nigeria [12]. Poor knowledge of the HbA1c has also been reported by Arslantas et al. [16]. A possible reason for this is unavailability or scarcity of this test in Nigeria at this time. While daily blood glucose monitoring tells how blood sugar is doing at a specific point in time (allowing necessary changes in medicine, food, and exercise), the HbA1c test show an individual’s glucose control and thus risk of complications [17]. It also identifies changes in response to alterations in management and therefore gives a picture of long term diabetes management success [17]. The result in the present study would therefore portray that a majority of Nigerian diabetics are unaware of their risk of developing complications, and in the dark in terms of the long term management of their condition. A resultant effect of this will be deficient coping strategies and in the long run poor quality of life.

Furthermore, the population that knew that diet soft drink should not be used to treat low blood glucose was lower than that reported in US by Murata et al. [6] (19.6% vs. 57%), but almost at par with the 21% reported by Odili, Isiboge and Eregie [12]. It is also lower than in an earlier Nigerian study which revealed that 53.8% knew how to manage hypoglycaemia [13] This shows a near total lack of awareness of this complication, which may stem from a lack of or poor education from the health team, as it has been shown that there is even a low knowledge of diabetes among healthcare workers who are expected to deliver health education to the community [18].

As regards diet, majority (67.9%) could not identify baked potato as the diet with the highest carbohydrate content, a far cry from the 82% [12] and 50% [6] reported previously. This is plausibly due to the fact that Plateau state is the largest producer of potato in Nigeria and it is therefore one of the staple food in this area. It has been shown that potato may be beneficial to persons with diabetes because of its high fibre and manganese content, which aids in stabilizing blood sugar levels and reducing insulin resistance [19]. However, literature has shown that the processing of potatoes by boiling elicits lower glycaemic index (GI) and glycaemic load (GL) values when compared to frying, baking, and roasting [19,20]. The participants in this study will most likely be ignorant of this fact, considering “cooked” and “baked” to be no different from one another. It is pertinent that while educating patients on proper dieting; foods that are staple in these areas should be taken into consideration in terms of appropriate replacements, and difference in method of food preparation where applicable should be considered. This is because the method of cooking can alter the structure, and nature of the starches resulting in significant effects on postprandial blood glucose responses [21].

When asked what makes up a diabetes diet, only a slight majority answered correctly (50.5%), a figure lower than the 72% reported in a study among Pakistani Muslims [22]. While the later study was carried out in a developed country, Nigeria is a developing country where people in the process of trying to adopt and embrace the “Western lifestyle” do so with many misconceptions–one of them being that a the diet of most American people is the healthy way of eating.

### Sociodemographic determinants of diabetes knowledge

Knowledge increased exponentially as level of education, with those who had never attended school scoring lowest and those with tertiary education scoring highest. This is consistent with other studies in Kenya [23], and the US [15], but is at variant with other studies which reported no difference in knowledge with level of education [1,24,25]. Others have reported better knowledge among those who attained secondary education, possibly because majority of the participants were attending or had attended secondary education [12] and even among those who never attended school [10]. A possible reason why Nigerian studies report that the less educated were more knowledgeable than the more educated is because the questions in these studies sometimes require interpretation to the illiterate ones, therefore the way a researcher asked a question may have guided them to the right answer. It may therefore advisable to translate the questionnaire to the local dialect of the target population if the illiterate group is to be included in a study as this will provide a balanced means of analysing the effect of education on knowledge. Furthermore, since a more educated person may be more inquisitive while being counselled or educated on diabetes by a health professional than an illiterate, educators should be more proactive and tune up their pedagogical skills while dealing with the less educated to ensure maximal participation and assimilation.

Satisfaction with education received whether update courses or seminars was associated with good knowledge of diabetes. Satisfaction will mean incorporation of information received into their daily routine of dieting; lifestyle modification and prevention of injuries or deformities (e.g. foot protection), which will go a long way to improving their coping strategies and invariably quality of life. It will also improve their attitude towards diabetes, and in the long run change their practices to embrace healthier lifestyles [26]. Participants who either regularly read articles, or attended seminars on diabetes were more knowledgeable for obvious reasons. One of the most important being that traditional, time tested and outdated beliefs about diabetes will be done away with, paving way for embracing new and proven realization of the disease. A study in South Africa earlier reported that participants with counselling had better knowledge than those who had not received counselling [27].

Furthermore, the wealthy and those with intermediate income were shown to have better knowledge than the poor, a finding which is at variance with another Nigerian study [12] as well as that among Malaysian diabetics [1], but consistent with others among diabetics in Oman [28] and non diabetics in Malaysia [1]. Government and retired workers also performed better than the self and unemployed participants, a result which is at variance with Odili et al. [12] who found that occupation did not affect knowledge. A possible reason for the above findings is that the wealthy, intermediate, government and retired workers were the most educated; satisfied with education received; and read educational materials/attended seminars more often than the poor, self, and unemployed. Furthermore, the government and retired workers who were also the rich ones would have had more as well as regular access to educational materials through the internet, along with seminars organised in their workplaces of which the poor, and self and unemployed would not be privy to. To increase the level of awareness of diabetes to all and sundry therefore, free educational courses should be provided at the community level so as to reach out to the less advantaged also.

Age was also associated with knowledge, with participants within the age group of 51-60 scoring higher than others, a result almost in line with another study which reported best knowledge among the 40-59 years age group [27]. With the positive influence of education on knowledge in this study, this finding is probably explained by the fact that a majority of the participants in the above age group had attained tertiary education as compared to the others. This finding is at variance with other studies which have found younger age to be associated with better knowledge [12]. Older persons with diabetes tend to have less education, worse cognitive function, and more barriers to practicing appropriate self care than their younger counterparts with diabetes [29,30]. This reasoning may not have applied in this study because the diabetics aged 51-60 years either read more articles or participated in update courses and had better cognition of the education received than those of other age groups.

In this study there was no association between years since diagnosed of diabetes, having a family history of diabetes, type of diabetes and knowledge of diabetes. A finding at variance with other studies which have reported difference in knowledge by number of years with diabetes [10,12,27] and having a family history of diabetes [25,28]. While the above studies were carried out in a hospital setting, this study was community based. Nonetheless, our finding is consistent with other studies which found no difference in knowledge by duration of diabetes [31] and family history of diabetes [12]. A positive family history of a disease may affect one's level of perceived risk [32] and is the factor most significantly associated with the perceived risk of developing diabetes [33]. However, in a randomized controlled trial, Pierce et al. [34] found that family members of individuals with type 2 diabetes underestimate their own risk of developing the disease. This perceived risk will only be possible if an individual is aware of his family’s history of diabetes and in this study only a slim majority (51.6%) were privy to this knowledge. This gap can be bridged if educators encourage diabetic patients to intimate their progeny of the disease and inspire them to learn more about it.

A majority of the participants in this study knew that exercise is beneficial for diabetes and the figure was higher than that reported in another Nigerian study [13] (76.1% vs. 52.1%), but lower than the 92.6% reported among Pakistani diabetics [35]. However, the number of participants who exercised regularly in this study was higher than those reported by Tham et al. [25] (47.3% vs. 40%) and Okolie et al. [11] (47.3% vs. 7.3%). These results would probably portray that knowledge may not necessarily lead to good practice or performance; because even though they knew the benefits of exercise many of them did not participate in regular exercises. This may however be due to a number of factors that can either be modifiable (self efficacy and social support) or non-modifiable (age, sex, and race/ethnicity) [36-38]. While educating patients on the benefits of exercises, it is also crucial that modified factors hindering their regular participation should be identified and methods of tackling these impediments addressed pronto.

### Strengths and limitations of the study

The strength of this study is that it was carried out in a community setting, where diabetics of different backgrounds were represented. However, the study was conducted in a single centre and therefore limits the generalization of its findings. A larger sample would provide more power to detect significant relationships between the study variables and differences between groups.

## Conclusion

This cohort of Nigerian Diabetics had poor knowledge of diabetes, riddled with misconceptions owing to time tested and widespread traditional beliefs about the condition. They did not know a “diabetic diet”, “fatty food”, “free food”, effect of unsweetened fruit juice on blood glucose, treatment of hypoglycaemia and the average duration glycosylated haemoglobin (haemoglobin A1) test measures blood glucose. Age, level of education, regular attendance of update courses/reading updates regularly, satisfaction with updates received, employment, and level of household wealth were associated with good knowledge of diabetes. This study suggests that educators should be more proactive, while also tuning up their pedagogical skills so as to ensure maximal participation and assimilation especially for the less educated. Furthermore, free educational courses at the community level are justified so as to reach out to the less advantaged also.

## Competing interests

The authors declare that they have no competing interests.

## Authors’ contributions

USJ, MCO, BGO, OA and EBP conceived the study. USJ, BGO, OA and MCO carried out literature search, data analysis and wrote the first draft. USJ, BGO and MCO were involved in the study design. MCO, EBP, AUS were involved in data collection, abstraction and interpretation. All authors critically revised the manuscript for intellectual content. All authors have seen and approved the final manuscript.
